# The feasibility and acceptability of an early intervention in primary care to prevent chronic fatigue syndrome (CFS) in adults: randomised controlled trial

**DOI:** 10.1186/s40814-020-00595-0

**Published:** 2020-05-12

**Authors:** Hazel O’Dowd, Lucy Beasant, Jenny Ingram, Alan Montgomery, Will Hollingworth, Daisy Gaunt, Simon M. Collin, Sarah Horne, Beth Jones, Esther Crawley

**Affiliations:** 1 CFS/NHS Bristol, The Lodge, Cossham Hospital, Lodge Road, Kingswood, Bristol, BS15 1LF UK; 2 Bristol Medical School, University of Bristol, Bristol, BS8 1NU UK; 3 School of Medicine, University of Nottingham, Nottingham, NG7 2UH UK; 4 Bristol Randomised Trials Collaboration, Bristol Medical School, University of Bristol, Bristol, BS8 2PS UK; 5 CFS/NHS Bristol, Cossham Hospital, Bristol, BS15 1LF UK

**Keywords:** Chronic fatigue syndrome/myalgic encephalomyelitis (CFS/ME), Early intervention, Primary care, Feasibility, Randomised controlled trial

## Abstract

**Background:**

Chronic fatigue syndrome (CFS, also known as myalgic encephalomyelitis (ME)) is defined as fatigue that is disabling, is accompanied by additional symptoms and persists for ≥ 4 months. Treatment of CFS/ME aims to help patients manage their symptoms and make lifestyle adjustments. We do not know whether intervening early in primary care (< 4 months after onset of fatigue) can prevent the development of CFS/ME.

**Methods:**

This was a feasibility randomised controlled trial with adults (age ≥ 18 years) comparing usual care with usual care plus an early intervention (EI; a combination of psycho-education and cognitive behavioural therapy, CBT). This study took place in fourteen primary care practices in Bristol, England and aimed to identify issues around recruitment and retention for a full-scale trial. It was not powered to support statistical analysis of differences in outcomes. Integrated qualitative methodology was used to explore the feasibility and acceptability of recruitment and randomisation to the intervention.

**Results:**

Forty-four patients were recruited (1 August 2012–November 28, 2013), falling short of our predicted recruitment rate of 100 patients in 8 months. Qualitative data from GPs showed recruitment was not feasible because it was difficult to identify potential participants within 4 months of symptom onset. Some referring GPs felt screening investigations recommended by NICE were unnecessary, and they had difficulty finding patients who met the eligibility criteria. Qualitative data from some participant interviews suggested that the intervention was not acceptable in its current format. Although the majority of participants found parts of the intervention acceptable, many reported one or more problems with acceptability. Participants who discontinued the intervention or found it problematic did not relate to the therapeutic model, disliked telephone consultations or found self-reflection challenging.

**Conclusions:**

A randomised controlled trial to test an early intervention for fatigue in adults in primary care is not feasible using this intervention and recruitment strategy.

**Trial registration:**

International Standard Randomised Controlled Trials, ISRCTN72645894. Retrospectively registered on 17 May 2013

## Introduction

Fatigue lasting more than 1 month is reported by 10–24% of attendees in general practice [1–3]. The population prevalence of chronic fatigue syndrome/myalgic encephalopathy (CFS/ME), where fatigue has persisted beyond 4 months, is 0.2–2%. CFS/ME is a significant health burden, has a poor prognosis in adults and consumes considerable health resources [4]; however, it is not known whether early identification and treatment of disabling fatigue can prevent the onset of CFS/ME.

A study that explored the pathway from glandular fever to CFS/ME found that 9.4% of (*N* = 234) participants were chronically fatigued at 3 months, and 7.8% of participants met the criteria for CFS/ME at 6 months [5]. The conclusions indicated areas for early intervention based on addressing particular characteristics, including anxiety, depression, somatization and perfectionism [5]. Addressing these ‘predisposing’ factors using cognitive behavioural approaches could reduce the probability of a patient with disabling fatigue lasting 1–4 months becoming the debilitating long-term condition that is CFS/ME.

Evidence to support early intervention was provided by a small (*N* = 69) randomised controlled trial based in primary care, which suggested there were fewer cases of fatigue (odds ratio 0.31, 95% confidence interval 0.09–0.91) in patients randomised to a psycho-educational intervention compared to controls [6]. The psycho-education was based on a behavioural model of fear avoidance and suggests recovery may be delayed due to prolonged rest. This hypothesis is presented to the patient and a suggested activity plan is given to guide them on a graded re-introduction of physical activity. However, we do not know whether this approach translates to patients presenting with fatigue in general practice or whether it is only useful in those whose chronic fatigue has been triggered by glandular fever (or some other viral infection).

In this study, we report on the feasibility [7] and acceptability of recruiting participants into a randomised controlled trial of an early intervention (referred to subsequently as EI) for early-onset, disabling fatigue to inform the design of a full-scale trial comparing usual care with EI plus usual care. The aim of the study is to estimate study parameters including the feasibility of recruitment (including the number of eligible patients presenting to primary care), the acceptability of the intervention to patients and the completion of patient-reported outcome measure results.

## Methods

### Population

Practices were identified through the Avon Primary Care Research Collaborative, now called NIHR Clinical Research Network West of England. When a practice expressed an interest, their deprivation score was checked using the Public Health Observatories General Practice Profiles [8] to investigate how representative the sample of patients was. Practices across Bristol varied in their deprivation score which reflects population socio-economic status.

A target of 100 patients was set based on the fact that approximately a tenth of all GP consultations record a complaint of fatigue [1, 3]. Assuming each GP practices had a list of 10,000, this would suggest there would be 5000 appointments in each year per GP practice where fatigue was discussed [9]. We therefore assumed it would be feasible to recruit 100 patients in 12 months. Sample size calculations are not always appropriate in feasibility studies [10]; therefore, this number was considered adequate to estimate the parameters required to design a larger trial.

Adults were eligible for this study if they were aged over 18; reported fatigue for at least 1 month but less than 4 months; had known causes of fatigue, e.g. cancer had been excluded; had normal results for the screening blood tests recommended in National Institute for Clinical Excellence guidance for CFS [1]; and scored ≥ 4 on the Chalder Fatigue Scale [11].

### Patient recruitment

Eligible patients were identified by the consulting GP and were asked to consent to contact from the research team. If the patient was willing to find out more about the study, a researcher contacted him/her and arranged a visit at a convenient time and location (usually the patient’s home) to discuss and provide further information about the study, including the PIS (patient information sheet), the study rationale, the uncertainties about the effectiveness of either intervention, the known advantages/disadvantages of the interventions, the options available outside the study and the right not to take part or withdraw at any time and the consent forms. Patients who were willing to take part were randomised to either usual care or to usual care plus EI.

Data were requested from each practice on the consultation codes used during the period of the study. Consultations with the following codes on Egton Medical Information Systems (EMIS) were counted: tired all the time (TATT), fatigue, exhausted, malaise, and lethargy. EMIS records for a sample of the first 100 consultations from one practice from the date they entered the study were selected for more detailed analysis. The records were checked retrospectively, against the study eligibility criteria.

### GP recruitment

The research team stayed in regular contact with GP practices to remind them about the study and inform them when patients were recruited. A laminated flowchart for the study was given to each GP. Every GP practice was visited by a member of the research team at least once during the study period. A variety of prompts about the study were used including email, telephone call and flyers.

### Randomisation

Allocation was minimised by gender and age group retaining a probabilistic element using a computer-generated random number sequence and was implemented using an automated telephone randomisation service provided by the Bristol Randomised Trials Collaboration (BRTC) to ensure allocation concealment. We switched recruitment from 1:1 to 1:2 (in favour of EI) in April 2013 to increase the number of participants who would experience EI in order to investigate the acceptability of the intervention.

### Interventions

#### Usual care

All participants received usual care from their GP. Usual care is defined by NICE [4] as investigation for other causes of fatigue (including blood screen), symptomatic relief through pharmacology and referral on to specialist services where they exist. GP’s were asked to record the care given, including referral to other services. We recorded the number of participants referred to the regional specialist CFS/ME service.

#### Early intervention

Participants in the EI arm continued to receive their usual care from their GP. As part of the EI arm, they also received: an information booklet; one face-to-face treatment session (duration up to 1 h); and three telephone follow-up sessions (20 min each) at 2, 6 and 10 weeks. The intervention was started in the participants home by an experienced, trained CFS/ME therapist within 2 weeks of randomisation.

EI was adapted from treatment for CFS/ME delivered by the Bristol CFS/ME service, which follows NICE guidelines for CFS/ME [4]. EI is based on a cognitive behavioural model of fatigue, focusing on strategies to improve sleep (sleep hygiene) and to balance activity (using activity diaries). The intervention included making a sleep and rest routine, monitoring the type and amount of activity undertaken every day and helping to develop consistent daily activity levels. CBT was used to explore barriers to getting better including fearful cognitions, avoidance of perceived risky situations, all-or-nothing behaviour, inappropriate beliefs about rest and sleep and focusing only on symptoms as opposed to experiencing them as normal bodily sensations. Where possible, solutions were discussed with participants. EI treatment sessions were audio-recorded and reviewed by the PI to ensure adherence to the treatment manual.

#### Feasibility assessment

We assessed the feasibility of recruitment by assessing the number recruited and the percentage of potentially eligible participants recruited. We assessed the acceptability of recruitment by interviewing participants over the recruitment methodology. We assessed the acceptability of the intervention by interviewing participants about the views of the intervention as well as exploring the numbers that completed the intervention. We assessed the completion of the patient-reported outcome measures as an assessment of the acceptability of the patient-reported outcome measures.

### Patient-reported outcome measurement

The following inventories were completed by participants at their assessment with the researcher (baseline) and then at 12-week and 6-month follow-up: socio-economic status (education and employment), Chalder Fatigue Scale [11], visual analogue pain rating scale [12], SF-36 physical function sub-scale [13], the Hospital Anxiety and Depression Scale (HADS) [14], the EQ-5D-5L [15], an adapted 4-item Work Productivity and Activity Impairment and General Health (WPAI:GH) questionnaire [16], and a health resource-use questionnaire, developed for this study which asked questions about health service use and travel costs most relevant to the CFS/ME population.

Reminders were sent out if follow-up questionnaires had not been returned after 2 weeks, followed by a phone call to persistent non-responders at 4 weeks, during which questionnaires could be completed by telephone, with a member of the research team,

### Data analysis

We estimated the number of eligible patients from routinely recorded data. We recorded the number of potentially eligible patients who consented to contact, the number who were eligible, and the number who consented to the study, were randomised and those who declined. We conducted a descriptive analyses of the baseline characteristics (median and inter-quartile range). We described the group mean (95% confidence intervals) for the 6-month outcome in both groups. Analysis is presented of available cases following intention to treat (ITT) principles. No hypothesis tests were conducted because of the nature of the study as a feasibility study.

#### Sample size

As the aim of this study was to assess the feasibility of a future definitive trial, we did not undertake a formal sample size calculation

### Qualitative data collection and analysis

All recruitment consultations were routinely audio-recorded, to document the interaction between recruiter and potential participant to explore information provision, recruitment techniques, patient treatment preferences and randomisation decisions to identify recruitment difficulties and support change. In-depth interviews were undertaken with patients to determine the acceptability of the study methods and the interventions and with the GPs to determine the acceptability of the recruitment methods. Interviews were semi-structured following a checklist of topics to ensure consistency, but flexible enough to allow GPs and patients to raise issues of importance. Interviews lasted approximately 30 min and all were audio-recorded with consent, transcribed verbatim and anonymised.

Participants and GPs were given the option of a face-to-face or telephone interview. Participants were interviewed in their own home or GP surgery, and GPs were interviewed in their place of work. Some participants chose to provide brief telephone feedback (discussion lasting less than 10 min) about their experiences because they did not have time to discuss the full interview schedule. When participants did not have time to take part in an interview, they were asked to comment on their experiences of the interventions and where appropriate, their reasons for discontinuing the EI programme before the final session.

Qualitative data analysis was an ongoing and iterative process commencing soon after data collection and informed further sampling and data collection. Interview transcripts and observation notes were imported into NVivo where they were systematically assigned codes and analysed thematically to identify themes using techniques of constant comparison [17]. Individuals exhibiting contrasting attitudes (‘negative cases’) were studied in detail to understand reasons underlying such contrasts and to gain a deeper understanding of the data and findings.

Recruitment to study consultations were purposefully selected for analysis at regular time points during the study, according to whether or not the study participant accepted randomisation, and those that highlighted issues of study acceptability (intervention cross over and study withdrawal) were also targeted for further analyses. They were analysed for content and presentation of information relating to the interventions using techniques of content analysis [18]. Two members of the research team analysed approximately 10% of the qualitative data independently to compare coding and enhance reliability. Descriptive accounts were produced, and theoretical explanations for behaviours, opinions and decisions developed.

### Serious adverse events (SAEs)

Serious adverse events were reported by clinicians from the specialist clinical team, primary care or members of the research team to the PI and the sponsor within 24 h. All SAEs were reviewed by the Research and Innovation Department at North Bristol NHS Trust.

### Ethical review

A *favourable* opinion was obtained in June 2012, by the South West 2 Local Research Ethics Committee.

At the end of November 2012, LREC approved a substantial amendment. This amendment allowed patients to be informed about the study before their blood results came back. In December 2012, information regarding the new recruiting pathway was sent to GPs. This asked GPs to provide patients with an informational leaflet at the initial contact and then again once a normal blood result was obtained. It was hoped this would improve recruitment.

## Results

Between July 2012 and December 2013, we recruited 14 GP practices to this study of which 13 practices referred patients into the study. The 14 GP practices were spread across all deprivation scores.

**Fig. 1:**
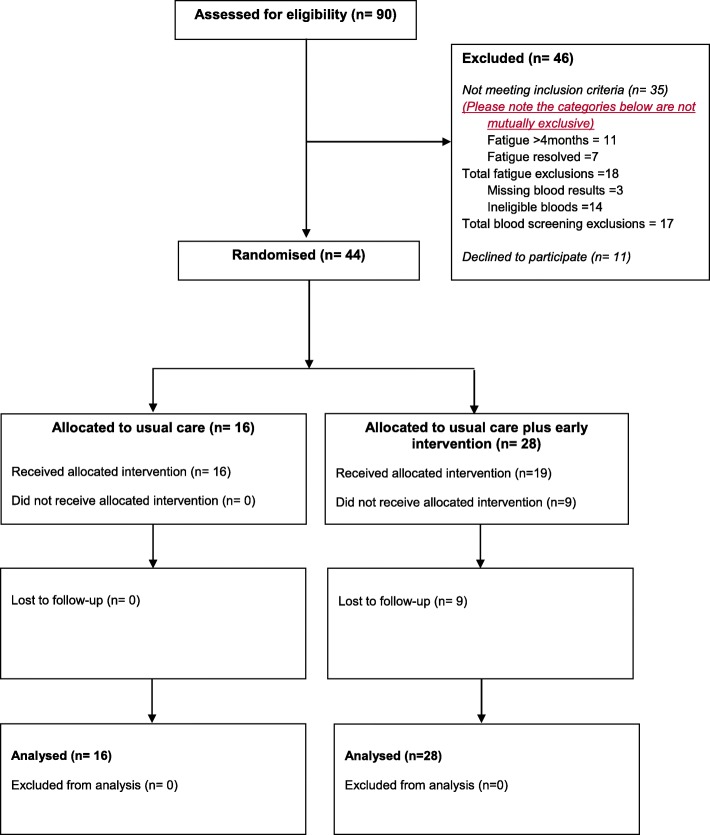
CONSORT early intervention in fatigue study

Of 90 potentially eligible patients referred to the study (Fig. 1), 44 patients were recruited 11 either did not respond to the invitation in the patient information sheet (PIS) or were not given a PIS by the GP. See additional file 2 for the CONSORT 2010 checklist of information to include when reporting a feasibility study. Forty-six patients did respond but were ineligible: 11 had been fatigued for > 4 months; 7 had fatigue which had resolved by the time they were approached about the study; 14 had abnormal investigations (13 abnormal blood results and one had a positive pregnancy test) which excluded a diagnosis of CFS/ME; three had not had all their blood tests completed. Of the 13 with abnormal blood test results, the most common reason (*n* = 7) for exclusion was raised inflammatory markers. Of the remaining six, one was anaemic; one had high sodium; one had a raised haemoglobin, urea, creatine kinase, and creatinine; one had high tissue transglutaminase antibodies suggesting coeliac disease; one had raised thyroid-stimulating hormone (TSH); and another had raised blood glucose levels (diabetes).

Of the 44 patients randomised, 16 patients were allocated to usual care and 28 patients were allocated to usual care plus EI. Of those patients allocated to EI, 19/28 attended 4 sessions, and 9 were lost to follow-up (2 at session 1, 2 at session 2, 3 at session 3, 2 at session 4). There were no losses to follow-up in the usual care arm.

We wanted to understand whether the problem with recruitment was because there were no eligible patients. We therefore explored this question by examining how many patients consulted their GP with fatigue. Data was provided by 11 of the 14 practices involved over the period of the study. Between April 2012 and 30 June 2013, there were 1711 consultations which included the codes: TATT/fatigue/malaise/lethargy (see additional file 1).

### Lack of compliance with the treatment arm

All 28 participants accepted the allocation at the randomisation appointment. 16 usual care and 28 EI received the treatment as allocated. Table 1 (Baseline characteristics) shows that the EI treatment group were slightly older (median age 40.0) than the control group (median age 37.5) and had a lower proportion of females (68% compared with 88%). Those allocated EI also had a lower median SF-36 physical function score (67.5 compared with 70.0), higher median pain score (32.5 compared with 22.5), higher median HADS depression score (8.5 compared with 6.5) and lower median HADS anxiety score (8.5 compared with 10.5). Chalder Fatigue scores were similar in both groups.

**Table 1:** Baseline characteristics

Baseline characteristics of the randomised population	Treatment group	
	Control (***n*** = 16)	Intervention (***n*** = 28)
**Demographic data**		
Median (25th, 75th centiles) age	37.5 (29.5, 50.0)	40.0 (32.0, 47.0)
Number female (%)	14 (88)	19 (68)
Number white (%)	16 (100)	26 (93)
**Clinical data (median (inter-quartile range))**		
SF-36 physical function score	70.0 (57.5, 95.0)	67.5 (42.5, 80.0)
Median Chalder Fatigue score (25th, 75th centiles)	24.5 (21.0, 26.0)	23.0 (20.0, 28.0)
Median pain VAS (25th, 75th centiles)	22.5 (0.0, 61.0)	32.5 (7.5, 62.5)
Median HADS Anxiety score (25th, 75th centiles)	10.5 (6.0,14.5)	8.5 (6.0, 12.0)
Median HADS Depression score (25th, 75th centiles)	6.5 (5.0, 9.0)	8.5 (4.5,10.5)

While the median EQ5D-5L were similar between the two groups, the EI treatment group had a lower percentage in paid work (64% compared with 81%), but similar hours missed from work over the past 7 days due to health problems and more hours missed from work over the past 7 days due to other reasons (median 1.0 intervention compared with 0 h control). These data can be found in Table 2.

**Table 2:** Baseline economic characteristics

Baseline economic characteristics of the randomised population	Treatment group	
	Control (***n*** = 16)	Intervention (***n*** = 28)
**EQ5D-5L**		
Median EQ5D-5L (25^th^, 75^th^ centiles)	0.710 (0.581, 0.790)	0.716 (0.491, 0.811)
**WPAI: GH 1**		
Number in paid work (%)	13 (81)	18 (64)
Median hours missed from work due to health problems in the past 7 days (25^th^, 75^th^ centiles)*	0.0 (0.0, 4.0)	0.0 (0.0, 2.0)
Median hours missed from work due to other reasons in the past 7 days (25^th^, 75^th^ centiles)*	0.0 (0.0, 7.5)	1.0 (0.0, 15.0)
Median hours worked in the past 7 days (25^th^, 75^th^ centiles)*	30.0 (10.0, 40.0)	24.5 (16.0, 38.0)
Median health problems affected productivity while working in the past seven days (25^th^, 75^th^ centiles)**	4.0 (3.0, 7.0)	4.0 (2.0, 6.0)
Median health problems affected productivity other than working in the past seven days (25^th^, 75^th^ centiles)	6.0 (5.0, 8.0), *n*= 15	5.5 (3.5, 8.0)

*Only answered by those patients who are currently in paid work (13 in the control group, 18 in the intervention group)

**Only answered by those patients who are currently in paid work and worked more than 0 h in the past 7 days (11 in the control group, 17 in the intervention group).

### Outcomes

Return of follow-up questionnaires was generally low but was marginally better at 6 months (primary time point, see Table 3, 36/44 returned) compared to 3 months when only 35/44 returned any questionnaires and only 34/44 (77%) returned the SF-36 physical function sub-scale. We have not presented the 3 month outcomes because we wanted to analyse the feasibility of collecting 3-month outcomes for future mediation analyses in a full trial. Table 3 shows the change in fatigue, pain, physical function (SF-36 physical function sub-scale), anxiety, depression, quality of life and loss of earnings data at 6 months. There was a reduction in mean Chalder Fatigue score at 6 months in both groups (control 14.7, intervention 12.3). There was no evidence of any difference between the two treatment groups in any of the clinical outcomes, but no statistical tests compared the differences between the two groups on this small sample of feasibility data.

**Table 3:** Six-month clinical and health economic outcomes

6-month clinical and health economic outcomes	Control group mean (95% confidence interval ), ***n***	Intervention group mean (95% confidence interval ), ***n***
Chalder Fatigue score	14.7 (10.6, 18.9), 16	12.3 (9.3, 15.3), 20
Pain VAS	18.9 (5.8, 31.9), 16	22.1 (8.5, 35.7), 19
SF-36 physical function score	84.4 (72.4, 96.3), 16	76.8 (65.1, 88.4), 20
HADS Anxiety score	7.8 (4.9, 10.6), 16	7.8 (5.4, 10.3), 19
HADS Depression score	4.6 (2.9, 6.2), 16	4.9 (3.0, 6.9), 19
EQ-5D-5L score	0.77 (0.677, 0.870), 16	0.749 (0.643, 0.855), 19
h missed from work due to health problems in the past 7 days	1.8 (− 0.4, 4.1), 11	5.3 (− 2.5, 13.1), 15
Health problems affected productivity while working in the past 7 days	3.0 (1.6, 4.4), 12	1.8 (0.6, 3.0), 13
QALY	**Control group AUC (SD) (*n*= 16)**	**Intervention group AUC (SD) (*n*= 20)**
	0.371 (0.326, 0.416)	0.346 (0.293, 0.400)

There were no differences between the two groups in mean number of hours missed from work due to health problems or health problems affecting productivity.

### Patient note review

In the 14 recruiting practices, 1711 patients were recorded as being tired all the time (TATT) or presenting with fatigue, exhausted, malaise, and lethargy between 1 April 2012 and 30 June 2013 (see additional file 1). In the more detailed review, of the 100 records on EMIS sampled retrospectively, 56 out of the 100 analysed, met criteria for eligibility, as they did not have any other exclusionary medical or psychological diagnosis to explain the fatigue which had lasted between 1 and 4 months, however only 2 patients were recruited into the study. Of the remaining 54 patients who appeared to be eligible but were not recruited, one later developed CFS and one developed post-viral fatigue syndrome.

### Qualitative research findings

#### Recruitment consultations

Feedback was provided for the recruiter after the first five recruitment consultations were analysed. Consultations were good in terms of pitching of information, pace, invitation of questions, and the building of rapport, and patients were accepting of the randomisation process and their allocated intervention arms. The research team was able to highlight areas where information provision could be more balanced to promote equipoise between treatment arms and support changes to the way that information was presented and discussed with potential participants. Instead of using statements such as the following:

Recruiter: When people come into the study and they either get the therapy or they just see the doctor for their usual care

Information about the usual care arm was provided, in addition to information about the early intervention:

Recruiter: Already GPs are able to do quite a lot of things to help, they can run blood tests and exclude other medical problems, they can refer onto other specialists, they can help people with ways of managing day-to-day

Instead of highlighting difficulty or the fact that some participants would ‘just’ see the doctor, the recruiter was encouraged to promote equipoise and equality between study arms by highlighting the fact that there is currently a lack of evidence and we do not know if early intervention is necessary or beneficial to patients with early fatigue:

Recruiter: But we don’t know if an early intervention will help ....... there is a chance say, by trying to look at things early, it may be too early, and it may have no effect or in some cases it may make some people worse, we just don’t know

### Participant feedback

Out of forty-four participants randomised, 20 participants were interviewed, 8/16 in the usual care arm and 12/28 in the EI arm. A further 10 participants gave brief telephone feedback, (discussion lasting less than 10 min) about their experiences because they did not have time to discuss the full interview schedule (three in the usual care arm and seven in the early intervention arm). Twenty participants chose to discuss the study via telephone, eight were interviewed in a place of their choice and two provided feedback via email. Seventeen participants gave feedback 3 months after recruitment and thirteen gave feedback 6 months after recruitment. All 9 participants who dropped out of the intervention arm were contacted; seven provided feedback on why they had discontinued sessions and two did not respond to contact.

### Study documentation

Participants in both study arms found study documentation and processes acceptable. These participants were happy with the amount of information they had been given, felt it was understandable and clear, it didn’t contain lots of ‘jargon’ (participant 11: male, < 40 years, early intervention). One participant with dyslexia found the information sheet ‘daunting’ (participant 10: female, < 40 years, usual care).

Participant 2: Well I understood it, (information sheet) so if I can understand (laughs) yeah, yeah, yeah. No, I mean, you know, I could understand it all fine, not a problem. I don’t think you can give too much information (female, > 40 years, usual care)

All interviewed participants were happy with the information provided by the trial manager at the face-to-face recruitment to study session. Participants discussed the informed consent process as an opportunity to gain a more in-depth introduction to the study and valued the opportunity to ask further questions. Participants seemed to engage and relate to the purpose of the study, including the possible benefits that the study could have for them. Using questionnaires at the recruitment meeting helped participants identify their symptoms giving them a sense that these symptoms were legitimate.

Participant 6: Um yeah I was quite encouraged because um I think it helped to articulate the deficit in energy. Rather than thinking of, ‘Oh I’m tired all the time,’ um it was good to think about sort of what is different, what doesn’t work quite as well? (female, > 40 years, usual care)

### Interventions

Most participants (twelve) found parts of the intervention acceptable, but fifteen reported one or more problems with acceptability. Parts of the intervention which participants found positive and useful included the fact that it encouraged self-reflection, the identification of areas in their life where they may be putting themselves under unnecessary pressure and restoring balance in their work and home life.

Participant 11: In that space of time (while completing the intervention) um I was consciously looking at myself because of the study, I have to concede…and um – and all for the better. And not only that, I’ve stayed with it as well. (male, < 40 years, early intervention)

Participants felt that the intervention gave them ‘tips’ and ‘tools’ they could take forward and use in the future. However, the timing of the intervention in each participant’s illness varied, some felt they had already started to make these positive changes themselves before they started the intervention, these participants were unsure if they could attribute reduction in fatigue to the intervention. A small minority identified the techniques used as cognitive behavioural therapy (CBT).

Participant 9: Um and obviously it’s then hard to know whether it’s the study that’s had that impact or whether it was a time thing and it would have – it would have sort of cleared itself away. (female, < 40 years, early intervention)

Others felt they were still experiencing fatigue when the intervention came to an end, and two felt that they were ‘left’ to manage their symptoms on their own when the intervention ended.

Participant 23: Now I’ve had my last sort of chat with (early intervention practitioner) um, you know, my issues are still going on, albeit not as bad as what they were, but now I have no contact with anyone so I’m sort of out on me own with it now. (male, > 40 years, early intervention)

Participants who discontinued the intervention or found it particularly problematic did not relate to the therapeutic model, disliked telephone consultations or found the concept of self-reflection challenging.

Participant 25: I’ve not received any treatment (in the early intervention arm) the psychology pamphlet didn’t have any practical application to my role (work) or life... if you have heavy lifting work then you need to do get someone else who is younger to do it. It relied heavily on mood making; if you feel like that, try to change the way you feel by doing this, so you feel different kind of thing (male > 40 years, early intervention)

Participant 1: The one-to-one session at the surgery was ok, but the ones on the telephone were no good so I didn’t want to carry on with it (male > 40 years, early intervention)

Participants suggested that the intervention could be improved by making it more consistent and tailored to the individual and that it could be delivered more flexibly (e.g. by offering weekend and evening contact appointments).

Participant 15: I just didn’t feel it was consistent. I know, I know that she only had the 20 min to go through it with me...and I thought it was a really good plan, and it has helped me, but it hasn’t helped me as much as I hoped it would. ..but I think part of that reason is because it wasn’t tailored to the individual. (female > 40 years, early intervention)

Sixty-four per cent (18/28) of participants in the intervention arm were in paid employment, and some of those interviewed reported that arranging telephone follow-up sessions around their hours of employment was challenging.

Participant 17: It has been difficult arranging the phone consults. It has been difficult. Yeah, yeah I mean I’m not blaming (early intervention practitioner) at all. Um but that’s just how it’s worked out, it hasn’t worked out well for either of us to keep it on schedule (female > 40 years, early intervention)

There were mixed views about whether or not 20–30-min telephone intervention consultations were long enough.

Participant 11: They were set in stone 20 to 30 min, 30 min absolute max, sort of thing. And, yeah, if it slipped ever so slightly over 30 min that was down to me chatting probably. (laughs) But er – but yeah, no, they were – they were perfect really…I was thinking, ‘I ain’t sure I could make that last 20 min,’ but it was no problem at all. So – so yeah, so, you know, um the length of time was pretty good (male, < 40 years, early intervention)

Participant 15: I know what she was saying, and I know the advice, but because it was such – it was only a 20-min phone call, there wasn’t the opportunity…basically the phone calls needed to be longer (female > 40 years, early intervention)

The feedback from participants in the usual care arm of the study suggested variation in GP guidance in relation to symptom management and recognition of fatigue symptoms. One participant felt that their GP had taken their symptoms seriously, but some participants in the usual care study arm were disappointed that they were not receiving the early intervention programme and developed ways of managing their fatigue on their own. Two participants in the usual care study arm were later referred to the CFS/ME service by their GPs for further treatment.

Participant 6: I was disappointed to not get into the um – be selected for the other group. Um mainly because I think presenting to your doctor with this is quite difficult. I certainly felt I needed to be assertive in saying I’d like these tests, I’m not – you know, er I want something, er not – not necessarily drugs, but I want some exploration....I was heard but maybe not listened to initially (female > 40 years, usual care)

Participant 10: I’ve been taking amphetamine when I have to, to get me through the day …I went to the doctor’s for help but I just felt like I was brushed off. (female < 40 years, usual care)

### GP feedback

Qualitative interview data from GPs referring and recruiting to the early intervention study documented GP views of screening and study processes and explored the treatment options offered to patients presenting with early fatigue in primary care. Interviews were conducted with 12 GPs from 10 of the participating practices, representing a range of socio-economic areas, (three practices did not respond to requests to discuss the study and one practice declined).

The majority of GPs interviewed felt that the GP information sheet was acceptable and understood the recruitment process, 3 commented on the laminated flowchart being a useful point of reference. GPs reported a clear understanding of the study and their feeling that the study was worthwhile for patients experiencing short-term fatigue.

I mean I think the paperwork is really fine and easy to follow. And I think it’s a really good option for people, because often there’s not that much you can offer people (GP4 practice 11)

It was a very straightforward study to recruit into once you identified the patients (GP12 practice 13)

The majority (10/12) of GPs cited a general lack of patients fitting the study eligibility criteria as the main difficulty in referring the expected numbers of patients to the feasibility study for recruitment. The eligibility criteria were seen as limiting because the ‘window of opportunity’ that GPs had to identify and refer patients was too narrow (more than one, but less than 4 months of fatigue).

Yeah it’s not – it’s not – you know, tired all the time is a recognised general practice sort of symptom, but er, you know, I have been surprised that I haven’t seen any (GP5 practice 2)

All 12 GPs discussed the importance of blood screens to exclude physical causes of fatigue, but few of the GPs involved routinely carried out the full range of screening needed for the early intervention study. Only 1 GP said the study bloods were standard.

There’s a couple I wouldn’t necessarily have normally done probably...I mean I think everybody probably has their pet bloods they take for tiredness (GP2 practice 6)

We did have to tweak our – if we’ve got patients who are tired all the time or have fatigue, we have a set of bloods that we send them to the blood people for...and they didn’t exactly match yours. We don’t do all of the chronic fatigue bloods as a routine just for normal fatigue that’s just been a month, we wouldn’t (GP10 practice 10)

#### Study amendments to improve recruitment

In the first 3 months of recruitment, only 3 participants were recruited from 11 GP practices compared to the study target of 25 participants. To improve recruitment, we asked GPs to inform potential participants about the study at the GP appointment rather than waiting for normal blood test results, changed the randomisation ratio in favour of the EI, visited each practice, emailed weekly updates, telephoned each practice weekly and formally notified each practice when a patient was accepted into the study.

### Serious adverse events (SAEs)

One SAE was reported during this study which was not related to either intervention.

## Discussion

Despite fatigue being one of the most common complaints in general practice, this study shows that a trial designed to identify patients with fatigue and offer early treatment to prevent the development of CFS/ME is not feasible or acceptable in primary care using this methodology. GPs found it hard to recruit to the study because of difficulties identifying patients with fatigue between 1 and 4 months onset and difficulties with obtaining the correct screening blood test results. Not all patients found the treatment offered acceptable.

### Strengths and limitations

We recruited GP practices to represent the Bristol population. This means that the results from this study may not be generalisable to other regions in the UK or other countries. Demographic data on the economic and work status of the sample is provided in Table 2 (baseline economic characteristics). We incorporated qualitative research methods to enable us to investigate issues around randomisation and retention as well as to explore experiences of the early intervention treatment.

The level of fatigue documented by EMIS (1711 consultations within the study period) is consistent with previous studies [1, 2, 19, 20] which have suggested that 10–24% of patients attend their GP with fatigue as their primary symptom. In our study, only 90 patients were referred as potential participants. This could be for a variety of reasons including the fact that the majority of patients may have had fatigue for longer than 6 months (or less than 1 month), the difficulties GPs faced in identifying patients or a reluctance of patients to take part in the study. This is not consistent with one study [6] which successfully recruited patients after an episode of EBV and provided self-help advice on implementing a graded approach to exercise.

We considered recruiting participants after an infection but this would have limited the intervention to a small group of patients. We are not aware of previous studies that attempted to recruit participants with fatigue of 1 to 4 months duration. While it is possible that patients were not eligible, this is not consistent with our detailed notes review which suggested at least half of those not recruited were eligible. Problems with identifying patients in primary care for research trials has been highlighted in other disease areas [3, 21].

Not all the participants found the intervention acceptable. This is not consistent with previous trials in CFS/ME which have shown a high level of patient satisfaction and low dropout rates with similar treatment approaches [22]. This may be because the treatment sessions were much shorter (20 min) than the treatment sessions provided in specialist services (and trials testing effectiveness) which are normally 60 min long [23]. We noted that most of the participants were still in employment and that this is a different group of patients to those attending a specialist service where studies indicate over 50% of patients are unemployed [24]. Follow-up rates were relatively low but there is insufficient data to comment on whether the timing of follow-up was appropriate.

Some patients found attending the sessions difficult as they required appointments outside of work time and did not want follow-up phone calls. This may be because the early intervention needs to be adapted more for patients who are still working unlike those with CFS where many patients are no longer in work.

Participants expressed dissatisfaction about the management offered for their fatigue by their GP which is consistent with previous studies which have shown low levels of patient satisfaction in patients reporting chronic fatigue and chronic fatigue syndrome [25, 26]. There is also evidence of the difficulties GPs feel in diagnosing CFS/ME and dealing with patients that present with fatigue [27].

We were surprised that approximately a third of participants did not find the intervention acceptable. The reasons given for this include issues around the self-reflection required during the process and the participant not accepting the therapeutic model. It is possible that treatment developed for patients with long-term fatigue is not suitable or appropriate for those with fatigue lasting only 1 to 4 months, where a more traditional medical model may be more prevalent.

## Conclusions

CFS/ME is a significant health burden and preventing the development of CFS/ME is likely to result in significant savings to the NHS and improved quality of life for those it affects [4, 24]. Expecting GPs to recruit to studies brings challenges and Primary Care Research Networks (PCRNs), now Clinical Research Networks, were established to assist with recruitment by providing funding and sometimes personnel. Future studies should explore different recruitment mechanisms in GP surgeries including list searchers and using PCRN-funded staff to recruit patients as well as explore why GPs do not appear to feel that early intervention is helpful (and therefore refer into the study).

Further work needs to be done to develop an intervention that is appropriate for this group of patients using the methodology described in the MRC framework on developing and evaluating complex interventions [28].

## Supplementary information

**Additional file 1.** Patients coded TATT between 1st April 2012- 30th June 2013. Estimate of prevalence of fatigue in general practice during the period of the study.

**Additional file 2.** CONSORT 2010 checklist of information to include when reporting a pilot or feasibility trial.

## Data Availability

The authors had access to all the data. The corresponding author had full access to all the data in the study and had final responsibility for the decision to submit for publication. Given the nature of this dataset, access is controlled (DOI 10.5523/bris.3jgpsepshoqfo20gjrzadc56pu). Requests are referred to the University of Bristol Data Access Committee for approval before data can be released under an appropriate data access agreement.

## Abbreviations

CFS/ME: Chronic fatigue syndrome/myalgic encephalomyelitis
EBV: Epstein-Barr Virus
EI: Early intervention
GP: General Practitioner
