# Supplementary material for: The feasibility and acceptability of an early intervention in primary care to prevent chronic fatigue syndrome (CFS) in adults: randomised controlled trial
Source: Pilot Feasibility Stud. 2020 May 12;6:65. doi: 10.1186/s40814-020-00595-0 (PMC7216523; doi:10.1186/s40814-020-00595-0)
Supplement: Supplementary file 1 — Additional file 1. Patients coded TATT between 1st April 2012- 30th June 2013. Estimate of prevalence of fatigue in general practice during the period of the study. [file 40814_2020_595_MOESM1_ESM.docx]

We asked practices for data on the number of consultations with patients that were recorded as ‘tired all the time’ TATT or a simile. The details are presented below.

**Patients coded TATT between** **1^st^ April 2012- 30th June 2013**

| **GP practice** | **TATT consultations N= 1711** | **total number referred** | **total number recruited** |
| --- | --- | --- | --- |
| 1 | 382 | 13 | 4 |
| 2 | 139 | 3 | 1 |
| 3 | 248 | 4 | 0 |
| 4 | 24 | 6 | 4 |
| 5 | 17 | 7 | 5 |
| 6 | No data | 5 | 5 |
| 7 | 105 | 12 | 7 |
| 8 | 277 | 7 | 2 |
| 9 | 83 | 2 | 1 |
| 10 | No data | 0 | 0 |
| 11 | 191 | 7 | 3 |
| 12 | No data | 4 | 3 |
| 13 | 42 | 14 | 7 |
| 14 | 203 | 8 | 2 |
| **Total** | **1711** | **90** | **44** |

**Estimate of prevalence of fatigue in general practice during the period of the study**

Variable data were available on the number of patients presenting with fatigue and eligible for the study. We conducted a detailed retrospective review of the patient records for eligible participants over the recruitment period using a sample of 100 consultations in one of the practices. These data are presented below.

| Patients coded TATT and meeting eligibility | 56 |
| --- | --- |
| Not referred for the study | 54 |
| Referred to study | 2 |

Of the 54 patients not referred to the study three later received a diagnosis of CFS/ME, Post Viral Fatigue Syndrome or Fibromyalgia, and two already had a diagnosis of Irritable Bowel Syndrome, suggesting that presenting with fatigue was worth further intervention.
